# Inter-band Bispectral Analysis of EEG Background Activity to Characterize Alzheimer's Disease Continuum

**DOI:** 10.3389/fncom.2020.00070

**Published:** 2020-09-30

**Authors:** Aarón Maturana-Candelas, Carlos Gómez, Jesús Poza, Saúl J. Ruiz-Gómez, Roberto Hornero

**Affiliations:** ^1^Biomedical Engineering Group, Escuela Técnica Superior de Ingenieros, de Telecomunicación, Universidad de Valladolid, Valladolid, Spain; ^2^Centro de Investigación Biomédica en Red en Bioingeniería, Biomateriales y Nanomedicina, (CIBER-BBN), Madrid, Spain; ^3^Instituto de Investigación en Matemáticas (IMUVA), Universidad de Valladolid, Valladolid, Spain

**Keywords:** electroencephalography (EEG), bispectrum, Alzheimer's disease (AD), mild cognitive impairment (MCI), AD continuum, interactions

## Abstract

The aim of this study was to characterize the EEG alterations in inter-band interactions along the Alzheimer's disease (AD) continuum. For this purpose, EEG background activity from 51 healthy control subjects, 51 mild cognitive impairment patients, 50 mild AD patients, 50 moderate AD patients, and 50 severe AD patients was analyzed by means of bispectrum. Three inter-band features were extracted from bispectrum matrices: bispectral relative power (BispRP), cubic bispectral entropy (BispEn), and bispectral median frequency (BispMF). BispRP results showed an increase of delta and theta interactions with other frequency bands and the opposite behavior for alpha, beta-1, and beta-2. Delta and theta interactions, along with the rest of the spectrum, also experimented a decrease of BispEn with disease progression, suggesting these bands interact with a reduced variety of components in advanced stages of dementia. Finally, BispMF showed a consistent reduction along the AD continuum in all bands, which is reflective of an interaction of the global spectrum with lower frequency bands as the disease develops. Our results indicate a progressive decrease in inter-band interactions with the severity of the disease, especially those involving high frequency components. Since inter-band coupling oscillations are related to complex and multi-scaled brain processes, these alterations likely reflect the neurodegeneration associated with the AD continuum.

## 1. Introduction

Dementia due to Alzheimer's disease (AD) is a progressive neurological disorder that exhibits brain changes leading to cognitive and physical impairment. AD is the most common case of dementia, accounting for between 60 and 80% of all cases (Alzheimer Association, 2018). Some general symptoms are typically associated with AD, such as loss of short-term memory, behavioral changes, and problems with abstract reasoning, planning, and decision making (Alzheimer Association, 2018). Dementia progression is mainly divided in three severity stages: mild, moderate, and severe (Reisberg et al., 1982). Mild AD patients (AD_MIL_) are generally independent in a daily basis, requiring some assistance in order to ensure safety. They are often able to perform high cognitive tasks, such as driving, working or leisure activities. Subsequently, moderate AD patients (AD_MOD_) usually have difficulties with routine tasks and may exhibit confusion about time and place. AD_MOD_ patients tend to show the first behavioral changes, such as agitation and suspiciousness. Finally, in the last stage of AD, severe AD patients (AD_SEV_) gradually become unable to perform any day-to-day activity to a point where they are eventually completely dependent on others to survive. At this point, verbal communication is also limited (Reisberg et al., 1982). A transitional period between healthy cognition and early dementia is likely to occur. This stage is called mild cognitive impairment (MCI) (Petersen, 2004). MCI due to AD is considered a prodromal form of the disease since 15% of subjects with this condition develop AD per year (Davatzikos et al., 2011), while only 1–2% of people not suffering from any pathological cognitive decline begin to manifest dementia symptoms (Petersen, 2004). MCI is described with slight cognitive deficits, but insufficient to precise a dementia diagnosis (Petersen, 2004).

In order to help diagnose AD and MCI, electroencephalography (EEG) has been widely used (Vecchio et al., 2013). EEG is a non-invasive technique to measure the spontaneous electrical activity of the brain over time. A set of electrodes placed on the scalp acquires the voltage fluctuations generated by groups of synchronized neurons. EEG spectrum has been extensively demonstrated to be susceptible of reflecting dementia. EEG frequency-based measures, such as median frequency (Penttilä et al., 1985; Hornero et al., 2009) and spectral entropies (Abásolo et al., 2006; Maturana-Candelas et al., 2019), have been applied in this regard. Low values of median frequency and spectral entropy have been commonly associated with AD (Penttilä et al., 1985; Abásolo et al., 2006; Hornero et al., 2009; Maturana-Candelas et al., 2019). Other spectral parameters, such as alpha peak (McBride et al., 2014) or spectral flux (Poza et al., 2017), also showed significant differences between AD, MCI, and control groups.

Despite the invaluable potential of EEG spectral analyses to detect altered neuronal behavior, only power spectrum (PS) examinations are considered in most cases. A limitation of PS is its inability to measure non-linear interactions between frequency components. This issue is overcome by analyses that take higher order spectra (HOS) into account. HOS is defined in terms of higher order statistics, or “cumulants” (Nikias and Mendel, 1993). Whereas PS is the spectra of second-order cumulants, HOS of third-order cumulants is called bispectrum (Nikias and Mendel, 1993). Bispectrum is calculated through the Fourier transform of third-order statistics, where the skewness of the distribution of the series is reflected (Chua et al., 2010). This point is crucial, as it permits to reveal divergences from Gaussianity. This allows bispectrum to discern non-linear interactions, such as phase coupling, which are suppressed under PS analyses (Nikias and Mendel, 1993). Although information contained in the PS is frequently enough to describe statistically any temporal series (Nikias and Mendel, 1993), extracting information of non-linear elements can be decisive to elucidate physiological perturbations from biomedical signals.

Many studies have applied bispectrum to EEG data in order to characterize different diseases and cognitive processes. For instance, Yuvaraj et al. (2018) applied HOS to develop a diagnosis algorithm able to discriminate controls from patients with Parkinson's disease. In another study, an automatic epileptic seizure detector using HOS-based measures was designed (Chua et al., 2007). Bispectrum has been also employed to determine the depth of anesthesia by Bispectral Index (BIS) calculation (Rampil, 1998; Tiefenthaler et al., 2018). AD has been studied by means of bispectrum analyses as well. In fact, a significant decrease of BIS has been observed in patients with dementia (Renna et al., 2003; Spiegel et al., 2006). Bispectral methods have also been used for emotion assessment (Hosseini, 2012) and the analysis of short-term memory processing (Schack et al., 2002), suggesting that interactions between oscillators at different frequency bands are related with complex neuronal processes. Bressler (1995) remarked the importance of cooperation between different cortical areas to achieve complex brain operations. Since AD is widely known as a “disconnection syndrome” (Delbeuck et al., 2003), the study of inter-band interactions may therefore help to elucidate these disturbances in neocortical dynamics.

Previously, different bispectral features have been used to characterize EEG dynamics, such as the mean of bispectral magnitude (Nasrolahzadeh et al., 2018; Vaquerizo-Villar et al., 2018), sum of logarithmic amplitudes (Nasrolahzadeh et al., 2018; Vaquerizo-Villar et al., 2018), bispectral entropies (Wang et al., 2015; Nasrolahzadeh et al., 2018; Vaquerizo-Villar et al., 2018), or weighted center of bispectrum (Wang et al., 2015). These parameters were calculated from the triangular region that satisfies *f*_2_ ≥ 0, *f*_2_ ≥ *f*_1_, *f*_1_ + *f*_2_ ≤ *f*_*s*_, being *f*_*s*_ the sampling frequency of the signal (Chua et al., 2010). These values are sufficient to evaluate the bispectrum due to its symmetry conditions (Chua et al., 2010). However, the parameters calculated from this region as a whole are unable to describe interrelations between different frequency bands. To overcome this limitation, we propose three measures: bispectral relative power (BispRP), bispectral cubic entropy (BispEn), and bispectral median frequency (BispMF), calculated from the regions of the bispectrum that display the interactions between each band and the global spectrum. To the best of our knowledge, no previous study analyzed specific inter-band regions of the EEG bispectrum to characterize the AD continuum.

Based on the aforementioned considerations, and since complex cognitive processes are related with interactions between inter-band components, we hypothesize that aberrant physiological activity caused by dementia may be reflected in alterations of these interactions. Our aim is therefore to investigate whether the alterations of BispRP, BispEn, and BispMF are able to characterize the progressive EEG disturbances along the AD continuum.

## 2. Materials

### 2.1. Subjects

We analyzed the EEG from 252 subjects divided in five groups: 51 healthy control (HC) subjects, 51 patients with MCI due to AD, 50 AD_MIL_ patients, 50 AD_MOD_ patients, and 50 AD_SEV_ patients. Dementia and MCI due to AD were diagnosed on every subject following the criteria of the National Institute on Aging and Alzheimer's Association (Jack et al., 2018). Cognitive deficit for each subject was evaluated by means of the Minimental State Examination (MMSE) test (Folstein et al., 1975). For AD and MCI patients, an age older than 65 and a diagnosis from a specialized physician were required to be included in their respective groups. On the other hand, the exclusion criteria were the following: (i) presence of atypical signs of cognitive evolution, (ii) history of active or under treatment neoplasia, (iii) history of recent surgery, (iv) history of hypercatabolic states, (v) chronic alcoholism, and (vi) indications of vascular pathology. HC were also evaluated to assess whether they satisfied some requirements in order to participate in this study. The applied criteria were the following: (i) age older than 65, (ii) MMSE scores equal or higher than 27, and (iii) absence of history of neurological or major psychiatric disorders. All subjects and caregivers gave written informed consent to participate in the study, according to the recommendations of the Code of Ethics of the World Medical Association (Declaration of Helsinki). The protocol was approved by The Ethics Committee at the Porto University (Porto, Portugal). Table 1 shows the demographic data of the participants.

**Table 1:** Demographic data.

		**Age**	**Sex**	**MMSE**	**Education**	**Smoker**	**Diet**	**Alcohol**	**Diseases**
**Group**	**N**	**($\bar{x}\pm \sigma$)**	**(F:M)**	**($\bar{x}\pm \sigma$)**	**(Pr:Sc)**	**(Y:Ex:N)**	**(M:A:H)**	**(Y:N)**	**(C:S:H)**
HC	51	80.1 ± 7.1	25:26	28.8 ± 1.1	33:18	1:11:39	42:7:2	18:33	30:1:12
MCI	51	85.5 ± 7.3	36:15	23.3 ± 2.8	38:13	2:5:44	45:3:3	9:42	32:12:10
AD_MIL_	50	80.5 ± 6.9	29:21	22.5 ± 2.3	36:14	3:4:43	44:4:2	7:43	30:10:9
AD_MOD_	50	81.3 ± 8.0	43:7	13.6 ± 2.8	37:13	1:6:43	44:6:0	5:45	28:4:18
AD_SEV_	50	80.0 ± 7.8	43:7	2.4 ± 3.7	45:5	0:2:48	42:6:2	0:50	33:5:16

*$\bar{x}$, mean; σ, standard deviation; education: (Pr: primary education or below, Sc: secondary education or above); smoking habits: (Y: smoker, Ex: ex-smoker, N: non-smoker); dietary habits: (M: balanced/mediterranean, A: antidiabetic, H: hypocaloric); alcohol consumption: (Y: occasional drinker, N: non-drinker); clinical story: (C: cardiovascular, as arterial hypertension, high cholesterol, etc.; S: sensorimotor as visual impairment, abnormal gait, etc.; H: hormonal, as diabetes, thyroid dysfunctions, etc.). Clinical story describes prevalence of clinical issues in the sample. Several diseases can affect the same subject and can only count one of each type*.

### 2.2. EEG Recording

Five minutes of resting-state EEG data were acquired for each subject, while staying in a relaxed position with their eyes closed. In order to minimize artifact presence, EEGs were recorded in a noise-free environment. Researchers made sure to avoid drowsiness of the participants during the procedure. EEG acquisition was performed with a 19-channel Nihon Kohden Neurofax JE-921A EEG System at electrodes F3, F4, F7, F8, Fp1, Fp2, T3, T4, T5, T6, C3, C4, P3, P4, O1, O2, Fz, Cz, and Pz of the international 10-20 system. Sampling frequency was established at 500 Hz.

EEG data were converted to ASCII files and stored in a personal computer. A preprocessing procedure was conducted according to these steps (Núñez et al., 2017; Ruiz-Gómez et al., 2018b; Maturana-Candelas et al., 2019): (i) mean removal; (ii) finite impulse response (FIR) bandpass filtering with a Hamming window between 1.5 and 30 Hz; (iii) independent component analysis (ICA) to remove components associated with myographic, cardiographic and oculographic noise; (iv) segmentation into 5 s epochs; and (v) visual rejection of artifacted epochs. An average of 38.81 ± 13.03 (mean ± standard deviation) artifact-free epochs per subject were selected. Digital procedures in this study were carried out with MATLAB® (R2018 version, Mathworks, Natick, MA).

## 3. Methods

### 3.1. Bispectral Analysis

HOS describe the spectral properties of cumulants and moments of higher orders (Nikias and Mendel, 1993). Second-order spectra (PS, a function based on the signal autocorrelation in the time domain) methods are frequently used to characterize AD (Dauwels et al., 2010). However, they are unable of perceiving components that remain hidden because of their non-linear nature (Nikias and Mendel, 1993). Phase and amplitude coupling between frequency components of a signal is a common concept that cannot be measured by these conventional metrics. In this work, bispectrum (spectral representation of the third-order cumulant) is used to study interactions between different frequency bands. The bispectrum of a signal *x*(*t*) is defined as

(1) $$\begin{array}{r} Bisp\left(f_{1},f_{2}\right)=E\left[X\left(f_{1}\right)X\left(f_{2}\right)X^{*}\left(f_{1}+f_{2}\right)\right], \end{array}$$

where *X*(*f*) is the Fourier transform of the signal *x*(*t*), X^*^(*f*) its complex conjugate, and *E*[·] corresponds to the expectation operation (Chua et al., 2010). As a result, a bispectrum matrix is obtained, representing the interactions between each pair of frequency components of the signal spectrum. In order to simplify analysis, a grand-averaged matrix across all epochs and channels was obtained for each subject. The resulting bispectrum matrix was normalized before any further procedure.

### 3.2. Bispectrum Features

A wide variety of features extracted from bispectrum has been previously proposed, such as the mean of bispectral magnitude, the sum of logarithmic amplitudes, the bispectral entropy and the weighted center of bispectrum (Venugopal and Ramakrishnan, 2014; Wang et al., 2015; Nasrolahzadeh et al., 2018; Vaquerizo-Villar et al., 2018). These parameters quantify global interactions between all the spectral components. However, feature extraction is commonly calculated from the whole bispectrum matrix, ignoring particular interactions between frequency bands. To overcome this limitation, three new parameters, which measure the interaction of each frequency band with the global spectrum, are applied in this study: bispectrum relative power (BispRP), bispectrum cubic entropy (BispEn), and bispectrum median frequency (BispMF). Another novelty aspect of these parameters is the exclusion of the self-band elements located in the diagonal for each calculation, allowing the assessment of the interactions between each band and strictly the rest of the spectrum. This approach is not often considered when analyzing the bispectrum, and may provide new insights about the categorization of the AD. Bands of interest corresponded to the classical frequency bands: delta (δ, 1.5–4 Hz), theta (θ, 4–8 Hz), alpha (α, 8–13 Hz), beta-1 (β_1_, 13–19 Hz), and beta-2 (β_2_, 19–30 Hz). BispRP, BispEn, and BispMF were calculated for each band of interest. These three parameters describe inter-band coupling properties in a complementary way and may help to elucidate how AD affects to neural interactions. The algorithms are defined below:

- BispRP describes the amount of accumulated bispectral power of a specific band interacting with the rest of the spectrum. High values indicate that a certain band is associated with greater inter-band interactions. BispRP is defined as
  (2) $$\begin{array}{r} BispRP=\underset{\left(f_{1},f_{2}\right)\in \rho }{\sum }|Bisp\left(f_{1},f_{2}\right)|, \end{array}$$
  where ρ corresponds to the region of the bispectrum matrix that reflects interactions between a specific frequency band and the global spectrum, excluding the interactions among frequencies of that frequency band.
- BispEn describes how bispectral values are distributed in the region associated with a specific frequency band. BispEn is implemented in this study based on the Shannon definition of entropy (Chua et al., 2010). Homogeneous-distributed interactions result in high BispEn values, while those condensed in fewer components cause this parameter to decrease. BispEn is defined as follows:
  (3) $$\begin{array}{r} BispEn=-\frac{1}{N}\underset{i}{\sum }p_{i}ln\left(p_{i}\right), \end{array}$$
  where
  (4) $$\begin{array}{r} p_{i}\left(f_{1},f_{2}\right)=\frac{|Bisp\left(f_{1},f_{2}\right){|}^{3}}{\underset{\left(f_{1},f_{2}\right)\in \rho }{\sum }|Bisp\left(f_{1},f_{2}\right){|}^{3}}, \end{array}$$
  being *i* each point in region ρ and *N* the total number of points in ρ.
- BispMF is defined as the frequency at which the total spectral power of the bispectrum at ρ is halved. BispMF indicates the tendency of a frequency band synchronizing more with higher or lower frequency components of the global spectrum. BispMF is defined below:
  (5) $$\begin{array}{r} 0.5\underset{\left(f_{1},f_{2}\right)\in \rho }{\sum }|Bisp\left(f_{1},f_{2}\right)|=\overset{BispMF}{\underset{\left(minf_{2}\right)\in \rho }{\sum }}\underset{f_{1}\in \rho }{\sum }|Bisp\left(f_{1},f_{2}\right)|. \end{array}$$

### 3.3. Interpretation of Bispectral Features

**Figure 1:**
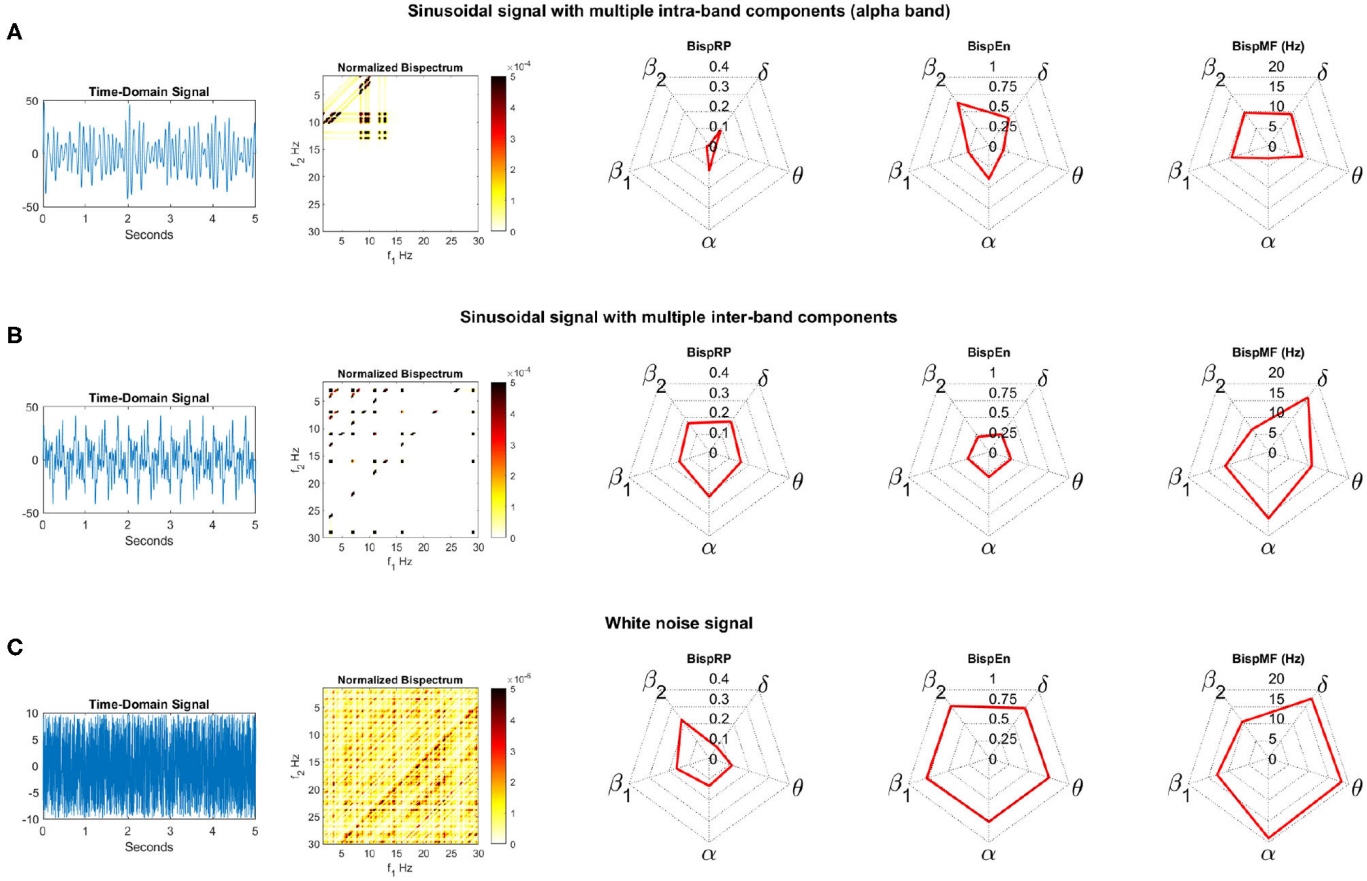
Time-domain synthetic signals with bispectrum values and BispRP, BispEn, and BispMF values for each band. **(A)** Sinusoidal signal with multiple intra-band components (alpha band). **(B)** Sinusoidal signal with multiple inter-band components. **(C)** White noise signal.

In order to facilitate the understanding of the proposed features (BispRP, BispEn, and BispMF), these metrics have been applied to three synthetic signals: (a) a sinusoidal signal with multiple intra-band components (sine waves at 8.5, 9.4, 10, 12, and 12.9 Hz); (b) a sinusoidal signal with multiple inter-band components (sine waves at 3, 7, 11, 16, and 29 Hz); and (c) a white noise signal. Bispectrum, along with the aforementioned bispectral features, are represented in Figure 1. Due to the large differences between high and low values in the bispectrum, the visualization scale has been adjusted to better reflect the former. For this reason, white regions of the bispectrum correspond to very low values, but they are not necessarily zero.

For the sinusoidal signal with multiple intra-band components (Figure 1A) the bispectrum shows several interactions in the alpha region, as expected. Other inter-band components, located in delta/alpha region, appear due to the interaction between all the sine waves. Since intra-band interactions are discarded in this study (i.e., interactions within the 8–13 Hz area in which the most prominent peaks appear), the only bispectral values that are considered to calculate any feature are the values for the inter-band ranges. Thereby, BispRP values are very low because most of the power of the bispectrum is located in the alpha/alpha range, which is discarded for BispRP computation. High BispRP values are obtained at delta and alpha, due to the interactions between them. Regarding BispEn, it describes the distribution of inter-band interactions. BispEn values are thus rather low for delta, theta, alpha, and beta-1, which indicates that only a few interactions appear in each inter-band region. Beta-2 is the band with the highest level of entropy because no peaks are present in the region of interactions between beta-2 and the remaining frequency bands. Although beta-1 band does not seem to have any interaction as well, its proximity to the alpha band contributes to lower entropies. Finally, BispMF describes the median frequency component with which another band is interacting with. As expected, delta, theta, beta-1, and beta-2 bands interact with alpha given no other activity in the bispectrum is present.

The bispectrum corresponding to the sinusoidal signal with multiple inter-band components (Figure 1B) shows interactions across all the bispectrum regions. In this case, BispRP values are almost equally distributed along bands. Analogously, BispEn shows similar values in each band, which means a similar distribution of inter-band interactions. For this signal, BispMF shows the predisposition of each band interacting with frequency components between 10 and 15 Hz, where are located the highest bispectrum values. Thus, BispMF for delta, theta, alpha, and beta-1 bands is around these two values. However, beta-2 displays a much lower value due to the presence of meaningful interactions with delta and theta frequency bands.

Finally, the white noise signal (Figure 1C) presents a high number of interactions distributed on the bispectrum. For this reason, BispRP is small in delta (due to its narrow frequency range), obtaining the highest value at beta-2 (owing to its wide frequency range), and shows intermediate values for theta, alpha, and beta-1. BispEn is high and uniform for all bands, as no dominant interactions appear. Finally, BispMF values coincide approximately with the middle of the bispectrum, except beta-1 and beta-2 that obtain values in the range of alpha band.

### 3.4. Statistical Analysis

The statistical analysis was performed as follows. First, in order to evaluate normality and homoscedasticity of our results, Kolmogorov-Smirnov and Levene test were conducted. Neither BispRP, BispEn nor BispMF results met parametric assumptions. Statistical differences between consecutive AD severity groups were therefore assessed with Mann–Whitney *U*-tests. In addition, the False Discovery Rate (FDR) was used to deal with multiple comparisons problem (Benjamini and Hochberg, 1995).

## 4. Results

**Figure 2:**
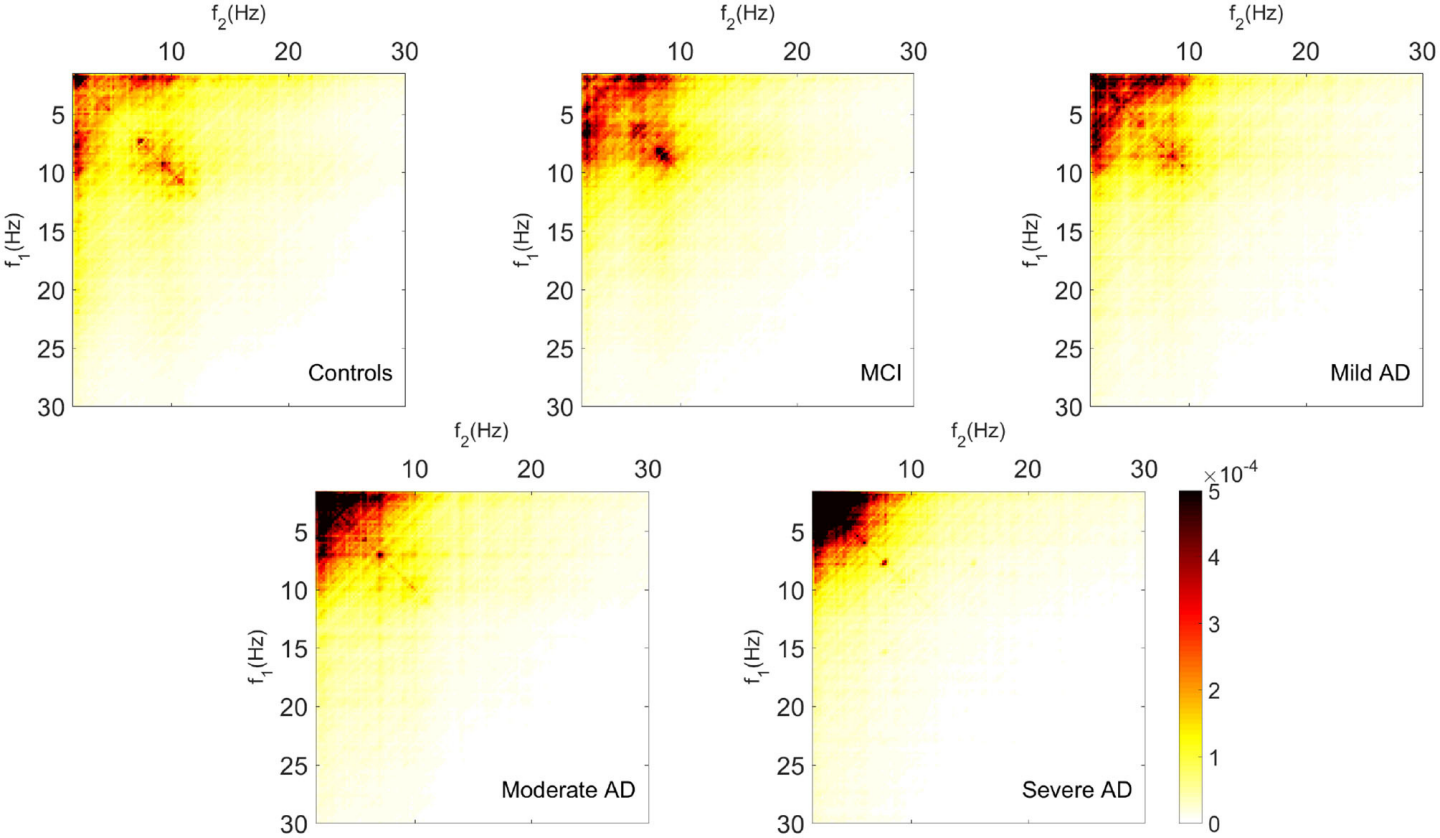
Grand-average of bispectrum values for each group.

Bispectrum and derived features were obtained for 51 HC subjects, 51 MCI patients, 50 AD_MIL_ patients, 50 AD_MOD_ patients, and 50 AD_SEV_ patients. Figure 2 displays the grand-averaged absolute values of bispectrum across channels for each group, showing a reduction of variety of inter-frequency coupling with the severity of the disease.

**Figure 3:**
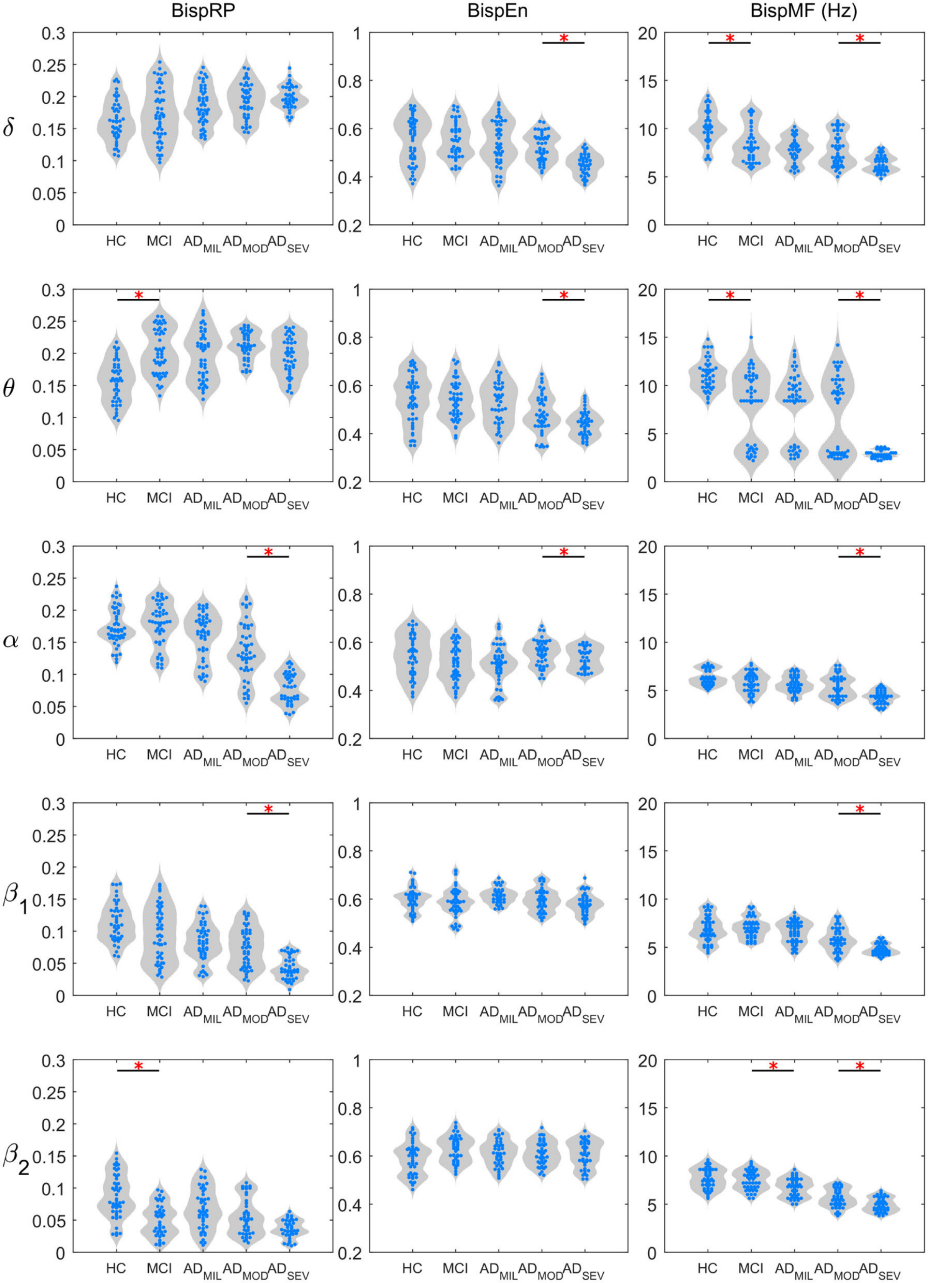
BispRP, BispEn, and BispMF value distributions on each band. Statistically significant differences between consecutive groups are indicated with a red asterisk (*p* < 0.05, FDR-corrected Mann–Whitney *U*-test).

The distribution of BispRP, BispEn and BispMF, for each frequency band, are represented in Figure 3. For this purpose, violin plots were employed. Statistical differences (FDR-corrected Mann-Whitney *U*-test) between consecutive groups are depicted on the top of each figure. Also, these values along with their respective *U*-values, are shown in Table 2. Increasing tendencies in BispRP can be observed in delta and theta bands in the AD continuum. On the other hand, BispRP decreases with the severity of the disease in alpha, beta-1, and beta-2 bands. Alpha and beta-1 bands showed statistically significant differences between the most severe groups (AD_MOD_ and AD_SEV_). In theta and beta-2 frequency bands, significant differences between HC and MCI subjects were also found.

**Table 2:** *U*-values and *p*-values from Mann–Whitney *U*-test for pairwise comparisons between consecutive groups (FDR-corrected).

	**Comparison**	**BispRP**		**BispEn**		**BispMF**	
**Band**	**(group vs. group)**	***U*-value**	***p*-value**	***U*-value**	***p*-value**	***U*-value**	***p*-value**
Delta	HC vs. MCI	2574.0	0.8087	2662.0	0.8429	**3106.5**	**0.0151**
	MCI vs. AD_MIL_	2402.0	0.3551	2729.0	0.5393	2670.5	0.7518
	AD_MIL_ vs. AD_MOD_	2452.0	0.7407	2661.0	0.5388	2727.0	0.3410
	AD_MOD_ vs. AD_SEV_	2506.0	0.9137	**2955.0**	**0.0188**	**2935.5**	**0.0233**
Theta	HC vs. MCI	**2098.0**	**0.0082**	2741.0	0.6047	**3021.0**	**0.0378**
	MCI vs. AD_MIL_	2701.0	0.6239	2766.0	0.4373	2681.5	0.7175
	AD_MIL_ vs. AD_MOD_	2347.0	0.4020	2700.5	0.4041	2657.5	0.5393
	AD_MOD_ vs. AD_SEV_	2791.0	0.1613	**2905.0**	**0.0378**	**2955.0**	**0.0188**
Alpha	HC vs. MCI	2522.0	0.6209	2739.0	0.6047	2803.0	0.4083
	MCI vs. AD_MIL_	2407.0	0.1085	2748.0	0.5048	2837.5	0.2501
	AD_MIL_ vs. AD_MOD_	2864.0	0.0712	2196.0	0.0743	2580.0	0.8087
	AD_MOD_ vs. AD_SEV_	**3171.0**	**0.0005**	**2902.0**	**0.0378**	**2959.0**	**0.0188**
Beta-1	HC vs. MCI	2921.0	0.1281	2735.0	0.6128	2581.0	0.8202
	MCI vs. AD_MIL_	2815.0	0.3267	2412.0	0.3879	2781.5	0.4020
	AD_MIL_ vs. AD_MOD_	2653.0	0.5393	2578.0	0.8087	2849.0	0.0767
	AD_MOD_ vs. AD_SEV_	**3102.0**	**0.0021**	2799.0	0.1484	**2985.0**	**0.0151**
Beta-2	HC vs. MCI	**3126.0**	**0.0126**	2285.0	0.0743	2581.5	0.8202
	MCI vs. AD_MIL_	2438.0	0.4373	2806.0	0.3410	**3033.0**	**0.0188**
	AD_MIL_ vs. AD_MOD_	2655.0	0.5393	2524.0	0.9972	2862.0	0.0712
	AD_MOD_ vs. AD_SEV_	2822.0	0.1117	2562.0	0.8429	**2949.0**	**0.0188**

*Comparisons with p-values below 0.05 are highlighted*.

BispEn displayed a decrease with the severity of AD in delta and theta bands, showing statistically significant differences between AD_MOD_ and AD_SEV_ groups. On the other hand, alpha, beta-1, and beta-2 did not show any clear tendency with AD progression.

Finally, BispMF displays a decreasing trend on all frequency bands along the progression of AD. Delta and theta bands exhibited statistical differences between HC and MCI. BispMF results in theta band showed a transition from upper-theta to under-theta frequencies. MCI and AD_MIL_, as well as AD_MOD_ and AD_SEV_ showed statistically significant differences at beta-2 band. Furthermore, significant differences for the AD_MOD_ vs. AD_SEV_ comparison were obtained in all frequency bands.

## 5. Discussion

Three bispectrum features (BispRP, BispEn and BispMF) were calculated for the five groups under study, from non-pathological elder subjects to severe cognitive-impaired AD patients. Our results suggest changes on interactions between EEG oscillators at different frequency bands in the development of the disease.

BispRP is the sum of the bispectrum values of each frequency band interacting with the global spectrum. Higher values of BispRP in a frequency band indicate more interactions between frequency components of that band with the others. As Figure 3 reflects, BispRP in delta and theta frequencies increases with the severity of the disease. This could be interpreted not just as an increase of coupled interactions between delta and theta with the rest of the bands but also as a reduction of interactions of the higher frequency bands with the global spectrum. On the other hand, alpha, beta-1, and beta-2 bands exhibited a BispRP decrease along the AD development, which may be related with a loss of coupling involving higher frequency components. The results from previous works exploring cross-frequency modulations on resting-state AD patients are consistent with these alterations (Fraga et al., 2014; Engels et al., 2016). For instance, a significant delta modulation decrease of beta frequency band and an increased delta modulation with theta band were reported, both intensified by the severity of the disease (Fraga et al., 2014). Furthermore, lower alpha/beta interactions have also been observed in AD (Palva and Palva, 2007; Fraga et al., 2014), being reported as signs of lower cognitive ability (Palva and Palva, 2007). Additionally, a decrease of cross-frequency coupling between beta band and all other bands was found in AD patients (Engels et al., 2016). This may suggest that interactions involving alpha and beta frequency bands are present in functional processes, which are lost throughout neurodegeneration.

BispEn was calculated to assess homogeneity of the distributions of interactions. As it can be observed, only delta and theta exhibited a decreasing tendency in entropy with AD progression, suggesting interactions of those bands with fewer components of the global spectrum. A decrease in EEG spectral irregularity is a widely known effect of neurodegeneration (Abásolo et al., 2006; Ruiz-Gómez et al., 2018a; Maturana-Candelas et al., 2019), which has been assessed by means of entropy analyses. Besides, an overall decrease of bispectral entropy was also observed in AD patients (Wang et al., 2015). Entropy is potentially capable to discriminate time series generated by different systems (Costa et al., 2005), which may be related to neural dynamic cooperation. Interestingly, our results indicate that these entropy alterations only occur at low frequencies, and loss of entropy in inter-band interactions at these frequencies is progressive in the AD continuum.

BispMF expresses which components of the global spectrum interacts predominantly with each frequency band of interest. Noteworthy, BispMF experimented a consistent reduction at each band as AD severity increases. The cause of these tendencies is two-fold. First, interactions involving higher frequency bands (alpha and beta) is diminished, and second, interactions between delta and theta are increased. This insight is consistent with our BispRP results, which show an increasing presence of delta and theta bands interactions with the global spectrum in more severe stages of AD. Alterations involving alpha and beta frequency bands have been previously related to AD common symptoms. For instance, theta/beta band coupling has been associated with reward/gain motivation (Putman et al., 2010), and theta/alpha interactions seem to be involved in retention of pictorial items (von Stein and Sarnthein, 2000). Changes in these cognitive capabilities are symptoms observed along the disease progression (Robert et al., 2006; Alzheimer Association, 2018). In fact, disturbances in these interactions have been used to develop a biomarker system to detect impaired cognitive states (Dimitriadis et al., 2015). Furthermore, decreases in delta/beta interactions were associated with behavioral perturbations (Schutter et al., 2006; Knyazev, 2007) and were also related to AD (Chow, 2000). According to lower frequency bands, previous research aiming to study delta/theta coupling reported an increase of these interactions in mouse models of AD (Wisor et al., 2005; Jyoti et al., 2010). These disturbances from normal EEG behavior were also strongly linked with increased amyloid beta deposition (Wisor et al., 2005; Jyoti et al., 2010); a decrease of BispMF and an increase of BispRP in delta and theta measures therefore seem a natural consequence of neurodegeneration.

In general, our results of EEG inter-band interactions indicate presence of neuronal interconnected systems and, thus, may help to elucidate multi-scaled brain processes from a physiological standpoint. Previous studies have assessed complex physiological mechanisms, such as attention (Palva, 2005) and learning performance (Canolty and Knight, 2010), in terms of cross-frequency synchronization. Further evidence has been reported about inter-band cross-frequency coupling in other brain processes, like working memory and reward stimulation (Palva, 2005; Cohen et al., 2009), suggesting these aspects to be associated with information processing and communication in large-scale brain networks (Canolty and Knight, 2010; Voytek, 2010). Bressler (Bressler, 1995) also pointed out that interareal synchronization (i.e., exact wave frequency and phase locking) is related with functional connections between cortical areas. This leads to think that different brain subsystems work synchronously so that more complex physiological processes can take place. In fact, loss of general EEG synchronization has been found in AD (Stam et al., 2003, 2005; Dauwels et al., 2010). Since this parameter has been linked with cooperation among diverse neuronal groups (Bressler, 1995), alterations in bispectral features may reflect impaired connectivity across the brain, in the form of neural pathways disruption by tissue alterations or neurotransmitter deficits (Tononi, 1998; Jelles et al., 1999; Jeong, 2004).

Although differences between five groups of progressive AD severity have been obtained, several issues must be taken into account in order to enhance the performance of this study. Firstly, differences in inter-band interactions may be much more evident when EEG is acquired under cognitive tasks. Cross-frequency coupling is especially visible when certain brain networks manage electrical activity of this kind. For instance, phase synchrony has been mainly studied in these circumstances (Palva, 2005; Pockett et al., 2009). For this reason, resting-state acquisition may have reduced the impact of these interactions. Secondly, since phase coupling is suggested to be related with activity at specialized brain regions, the obtained grand-average bispectrum values from the entire skull may diminish statistical differences in the calculated features. Previous research has appointed that specific channels may be more suitable to measure non-linear interactions (Fraga et al., 2014; Wang et al., 2015). Additionally, AD does not alter the entire brain but particular neural networks (He et al., 2008). In future works, we will aim to carry out further research specifying for local brain areas under diverse mental procedures and comparing the functional role of multiple frequency bands. Finally, resting-state neuronal rhythms have been demonstrated to be sensible to acquisition conditions, such as having the eyes open (Barry et al., 2007). Potentially, new frequency interactions could emerge by altering this factor. However, in this study EEG data were obtained exclusively from patients with the eyes closed. Studying the EEG from patients with the eyes open may expose new insights on neurodynamic behavior. Therefore, we will try to obtain new EEG recordings in this condition to conduct further investigation of the influence of neurodegeneration in inter-band coupling.

## 6. Conclusions

In this study, bispectrum and novel derived features were computed from EEG signals of MCI and AD patients. Our results suggest an overall decrease of spectral component interactions involving high frequency bands. This point is consistent with previous research aiming to relate brain functionality with inter-band synchronization. The underlying physiological phenomena of neurodegeneration processes could imply loss of communication between specialized neuronal groups. This may provoke the discussed tendencies on our bispectrum results with the progression of the disease. We can conclude that the analysis of inter-band interactions by means of bispectrum is able to characterize AD continuum.

## Data Availability Statement

The dataset is not publicly available due to some restrictions from the Ethical Committee. The data that support the findings of this study are however available on reasonable request from the corresponding author.

## Ethics Statement

The studies involving human participants were reviewed and approved by The Ethics Committee at the Porto University (Porto, Portugal. Report n° 38/CEUP/2018). The patients/participants provided their written informed consent to participate in this study.

## Author Contributions

AM-C, CG, and JP collected the signals. AM-C processed the signals, analyzed the data, and wrote the manuscript. CG and RH designed the study and interpreted the results. JP and SR-G interpreted the results. All authors have read and approved the final manuscript.

## Conflict of Interest

The authors declare that the research was conducted in the absence of any commercial or financial relationships that could be construed as a potential conflict of interest.
